# A Novel Universal Neutralizing Monoclonal Antibody against Enterovirus 71 That Targets the Highly Conserved “Knob” Region of VP3 Protein

**DOI:** 10.1371/journal.pntd.0002895

**Published:** 2014-05-29

**Authors:** Tanja K. Kiener, Qiang Jia, Tao Meng, Vincent Tak Kwong Chow, Jimmy Kwang

**Affiliations:** 1 Animal Health Biotechnology, Temasek Life Sciences Laboratory, Singapore, Republic of Singapore; 2 Department of Microbiology, Yong Loo Lin School of Medicine, National University of Singapore, Singapore, Republic of Singapore; University of North Carolina at Chapel Hill, United States of America

## Abstract

Hand, foot and mouth disease caused by enterovirus 71(EV71) leads to the majority of neurological complications and death in young children. While putative inactivated vaccines are only now undergoing clinical trials, no specific treatment options exist yet. Ideally, EV71 specific intravenous immunoglobulins could be developed for targeted treatment of severe cases. To date, only a single universally neutralizing monoclonal antibody against a conserved linear epitope of VP1 has been identified. Other enteroviruses have been shown to possess major conformational neutralizing epitopes on both the VP2 and VP3 capsid proteins. Hence, we attempted to isolate such neutralizing antibodies against conformational epitopes for their potential in the treatment of infection as well as differential diagnosis and vaccine optimization. Here we describe a universal neutralizing monoclonal antibody that recognizes a conserved conformational epitope of EV71 which was mapped using escape mutants. Eight escape mutants from different subgenogroups (A, B2, B4, C2, C4) were rescued; they harbored three essential mutations either at amino acid positions 59, 62 or 67 of the VP3 protein which are all situated in the “knob” region. The escape mutant phenotype could be mimicked by incorporating these mutations into reverse genetically engineered viruses showing that P59L, A62D, A62P and E67D abolish both monoclonal antibody binding and neutralization activity. This is the first conformational neutralization epitope mapped on VP3 for EV71.

## Author Summary

Over the last decade, EV71 has emerged as a major cause of severe hand, foot and mouth disease in the Asia-Pacific region, occasionally leading to fatal brain stem encephalitis in young children. The rapid progression and high mortality of severe EV71 infection makes it vital to identify neutralization epitopes and putative therapeutic monoclonal antibodies. In this study we mapped the first conformational neutralization epitope on the VP3 protein of EV71. This epitope was confirmed by introducing the mutations into reverse genetically engineered viruses which abolished neutralization with monoclonal antibody (mAb)10D3. The importance of this novel neutralization epitope lies in the optimization of putative EV71 vaccines because the VP3 knob could be incorporated together with VP1 into a bivalent subunit vaccine. Further, the universal recognition of a conserved site on EV71 VP3 and not CVA16 makes mAb 10D3 a valuable tool for differential diagnosis of hand, foot and mouth disease. An additional hope is that mAb 10D3 could be used as a therapeutic intravenous immunoglobulin (IVIG).

## Introduction

Human enterovirus 71 (EV71) is a causative agent of hand, foot and mouth disease (HFMD) which has become a serious health threat to young children in the Asia Pacific region over the last 15 years. Although HFMD is most commonly caused by members of the coxsackievirus family, which are genetically related to EV71, infection with EV71 is more often associated with neurological complications in children under 3 years of age and is responsible for the majority of fatalities [1]–[3]. A major concern has been the emergence of a syndrome of rapidly fatal pulmonary edema associated with brainstem encephalitis in the Asian epidemics [4]. In an outbreak of HFMD in 2008 in China, up to half a million cases were reported among children resulting in over 120 fatal cases, which were primarily due to EV71 infection [5]. A recent outbreak in Cambodia led to the deaths of 54 children, most of them under 3 years of age: All samples obtained from fatal cases tested positive for EV71. Since the nearly complete eradication of polio, EV71 is now regarded as the pre-eminent neurotrophic virus, and a threat to global public health [6], [7]. To date, there are no specific antivirals or vaccines for clinical use, and prevention is mainly achieved by disrupting virus transmission with improved public hygiene in kindergartens, preschools and daycare centers aided by the temporary closures of affected places.

A number of animal studies have shown that neutralizing antibodies stimulated by immunization with inactivated virus, VLPs, or displayed VP1, are cross-protective against heterologous strains and can passively protect mice and monkeys [8]–[14]. Further, studies on patients have indicated that EV71 infection is cleared by humoral immunity and clinical trials have shown the presence of neutralizing antibodies in the serum of immunized healthy adults and children [13], [15], [16]. This significant involvement of neutralizing antibody responses in the control of EV71 infection in humans would render IVIG treatment an ideal therapeutic agent to complement vaccination. Passive immunization by IVIG with pooled sera from convalescent human donors has been pioneered by Behring & Kitasato in the 1890's with the development of anti-diphtheria serum. However, besides the risk of transmitting human pathogens using pooled human sera, necessitating screening and treatment, there are other disadvantages, i.e. the availability of donors, batch to batch variability, and the presence in the serum of virus specific but non-neutralizing antibodies. A solution would be to exploit future passive immunotherapy based on monoclonal antibodies (mAb) produced in cell culture.

EV71 is a small non-enveloped single positive-stranded RNA virus belonging to the *Picornaviridae* family, genus *Enterovirus*, species Enterovirus A. The naked genome is enclosed in an icosahedral capsid composed of the four structural proteins (VP1, VP2, VP3, and VP4). While VP1-VP3 form the surface of the virion, VP4 is arranged internally [17], [18]. The rapid mutation rate of RNA viruses results in the emergence of new subgenogroups every few years. To date, 11 EV71 subgenogroups have been identified based on the VP1 capsid protein which is the most variable of the four [19], [20]. The three genogroups are denoted A, B and C, of which B and C are further divided into subgenogroups B1–5 and C1–5 [19]. Both the co-circulation of different genogroups and the emergence of novel strains have been observed in the Asian pandemics [21]–[24]. Interestingly, it has been shown that this genotyping does not correspond to the virus strain antigenicity which points to the presence of conformational epitopes, independent of sequence homology [25]–[29]. It is surprising then, that only 1 universal neutralizing epitope of EV71 has been described thus far, i.e. a linear epitope on VP1 which encompasses amino acids 215-KQEKD-219 [30]. This region is conserved among all EV71 subtypes - making it a universal neutralizing epitope - and a monoclonal antibody against this epitope is able to protect mouse pups from lethal EV71 infection [30]. Given that other picornaviruses such as poliovirus carry multiple neutralizing epitopes (including conformational epitopes), we expect EV71 to also possess more than one linear neutralization epitope [31]. Indeed, this is the case as we here report the isolation of the first conformational, universally neutralizing mAb against EV71 that is not on VP1.

## Materials and Methods

### Ethics statement

All animal experiments were carried out in accordance with the Guidelines for Animal Experiments of the National Advisory Committee on Laboratory Animal Research (NACLAR). Experimental protocols were reviewed and approved by Institutional Animal Care and Use Committee of the Temasek Life Sciences Laboratory, National University of Singapore, Singapore. (IACUC approval number TLL-IACUC-2013/002).

### Cell lines and virus stocks

African green monkey cell line (Vero ATCC number CCL-81) and Rhabdomyosarcoma cell line (RD ATCC number CCL-136) were obtained from American Type Culture Collection (ATCC) and grown in Dulbecco's modified Eagle medium (DMEM, Gibco, USA) containing 10% fetal bovine serum (FBS, Biowest, France) at 37°C with 5% CO_2_. Wild-type (wt) EV71 strains and CVA16 strain (U05876) were obtained from the Host and Pathogen Interactivity Laboratory, Department of Microbiology, Yong Loo Lin School of Medicine, National University of Singapore. The GenBank accession numbers of representative EV71 subgenogroups are listed in Table 1. Missing subgenogroups B1 (GenBank AF135901), B3 (GenBank AF376093) and C3 (GenBank AY125973) were constructed using the human RNA polymerase I reverse genetics system by inserting the relevant VP1 genes into the backbone of EV71 C4 strain Fuyang.Anhui.P.R.C/17.08/2 (GenBank EU703813). These viruses were propagated in RD cells cultured in supplemented with 2% FBS. Cell culture supernatants were harvested 4 days post infection (dpi), when 100% cytopathic effect (CPE) was observed. After three freeze-thaw cycles and filtration through a 0.2 um cut-off filter (Sartorius, Germany), aliquots were stored at −80°C. Virus activity was tested on RD cells in an end-point dilution assay to determine the 50% tissue culture infective dose (TCID_50_). For animal immunization EV71-B4 was inactivated with binary ethylenimine (BEI) for 48 h at 37°C as described by Bahnemann [32]. Virus was then concentrated 10-fold by ultracentrifugation at 100,000 g for 3 h and re-suspended in PBS.

**Table 1:** Representative strains of EV71 subgenotypes.

Subgenogroup	Name	GenBank	Neutralization Titer Mab 10D3
A	BrCr	U22521.1	2∧6
B1	RG EV71-VP1 (B1)	EU703813.1 backbone	2∧6
B2	7423/MS/87	U22522.1	2∧6
B3	RG EV71-VP1(B3)	EU703813.1 backbone	2∧6
B4	5865/SIN/000009	AF316321.2	2∧6
B5	NUH0083/SIN/08	FJ461781.1	2∧6
C1	Y90-3761	AB433864.1	2∧6
C2	NUH0075/SIN/08	FJ172159.1	2∧8
C3	RG EV71-VP1(C3)	EU703813.1 backbone	2∧8
C4	75-Yamagata-03	AB177813.1	2∧8
C5	3437/SIN/06	GU222654.1	2∧8

### Identification of EV71 specific mouse mAb 10D3 against EV71

Three specific pathogen-free BALB/c mice were immunized subcutaneously on days 0, 14 and 28 with inactivated EV71-B4 strain in 0.1 ml PBS, emulsified with an equal volume of adjuvant (Seppic, France). An intraperitoneal booster of the same virus dose without adjuvant was administered 3 days before the mice were euthanized and their spleen cells harvested. Splenocytes were then fused with SP2/0 myeloma cells as described [33] and the resulting hybridomas were grown in DMEM with 20% FBS containing HAT or HT for 10 days. The hybridomas were screened by IFA of Vero cells infected with EV71-B4 and cells secreting specific antibodies were subcloned by limiting dilution and cultured.

### Immunofluorescence assay (IFA)

Vero African green monkey kidney cells infected with EV71-B4 were used for antibody screening. Cells were seeded overnight into 96 well microtiter plates and infected with a 10^−6^ dilution of EV71-B4 the next morning. Upon observation of CPE, the cells were fixed in 4% paraformaldehyde (pH 7.4) for 20 min, and permeabilized with 0.1% Triton-X/PBS for 30 min. The cells were blocked with PBS containing 5% FBS for 1 h at RT and incubated overnight at 4°C with hybridoma cell supernatants or mAb 51 as positive control. Anti-mouse FITC-coupled secondary antibody was then added for 1 h at RT. The ells were washed three times with 0.1% PBS-Tween between each step. Results were documented with an inverted microscope (Olympus) with Nikon ACT-1 software. IFA of escape mutants and RG viruses was conducted with infected RD cells using the same protocol as above.

### Dot blot assay

Reverse-genetically (RG) constructed viruses were propagated in RD cell cultures. Harvested supernatants were purified by sucrose gradient centrifugation: cells were pelleted by centrifugation at 8,000× g for 40 mins, after which the supernatant was ultracentrifuged at 100,000× g for 2 h. The resulting pellet was re-suspended and centrifuged in a 20–60% discontinuous sucrose gradient at 100,000× g for 3 h, and the virus band was collected. To concentrate the virus, PBS was added to the sucrose gradient band which was further centrifuged at 100,000× g for 1 h, and resuspended in PBS. Protein concentrations were measured by Nanodrop, and all purified viruses were diluted to 0.3 mg/ml. 80 µl of RG viruses was then mixed with 20 µl of 10% SDS, boiled for 5 min, and diluted 10 times. 100 µl (around 30 µg) of virus was then dotted on a nitrocellulose membrane. The blot was blocked in 5% milk in PBS for 1 h at RT before incubation in primary antibodies 10D3 and 53 for 1 h at RT. After washing with PBS-T the blot was incubated with anti-mouse secondary antibody, then ECL reagent (GE Healthcare, USA), and the image captured by ChemiDoc MP imaging System (Bio-Rad Laboratories Inc. USA).

### Neutralization assay

The neutralization titer of mAb 10D3 (hybridoma cell culture supernatant) was measured in an *in vitro* microneutralization assay using RD cells. 100 TCID_50_ of wild-type, escape mutant, or RG viruses were mixed with an equal amount of 2-fold serial dilutions of mAb 10D3 or mAb 51 as a positive control. The mixtures were incubated for 1 h at RT before adding them in triplicates to the wells of microtiter plates containing 80% confluent RD cells. Presence of CPE was determined after 4 days by examination under the light microscope. The highest dilution of mAb that inhibited virus growth was considered the neutralizing titer and expressed as 2^x^. Assays were carried out independently three times.

### Selection of mAb 10D3 escape mutants

The wild-type virus stocks EV71-A, EV71-B2, EV71-B4, EV71-C2, and EV71-C4 were diluted to 50 TCID_50_×Neutralization titer against accordingly virus [34]. Then incubated in an equal volume of neat mAb 10D3 (hybridoma supernatant) for 1 h at room temperature (RT). The mixture was then transferred to 80% confluent RD cells in DMEM with 10% FBS and incubated for 4 days. If no CPE was observed, the supernatants were harvested, subjected to three freeze-thaw cycles and filtered with a 0.2 um cut-off before re-infecting a fresh batch of RD cells for 4 days. This was repeated until CPE was observed. 1–3 re-infection cycles were needed for CPE, and hence EV71 escape mutants to develop. The escape mutants were called E1–3/B4 (three individual experiments using EV71-B4 virus), E1–2/B2 (two individual experiments using EV71-B2 strain), E/A (EV71-A), E/C2 (EV71-C2), and E/C4 (EV71-C4). TCID_50_ was measured by end point dilution and IFA as well as microneutralization against mAb 10D3 was conducted to confirm abolishment of antibody binding and neutralization.

### Viral RNA isolation and cDNA sequencing

The viral RNA isolation kit (Qiagen, Germany) was used to extract viral RNA from filtered RD cell culture supernatants containing wild-type and escape mutant virus. Typical yields were 80–100 ng/µL as measured by Nanodrop (ThermoFisher Scientific, USA). Reverse transcription was carried out on 500 ng RNA, using gene- and strain-specific primers together with AMV reverse transcriptase (Roche Applied Science, Germany) according to the manufacturer's protocol. PCR amplification of 2 overlapping portions of P1 region was then conducted using the primer pairs (Table 2) and the High Fidelity PCR system (Roche Applied Science, Germany). The cycling parameters were as follows: Denaturation at 94°C for 2 min; followed by 10 cycles of denaturation at 94°C for 30 sec, touchdown annealing from 54°C to 45°C in 1°C decrements for 30 sec, extension at 72°C for 2 min; followed by 30 cycles of denaturation at 94°C for 30 sec, annealing at 45°C for 30 sec, extension at 72°C for 2 min+5 sec per cycle increments, and a final extension at 72°C for 7 min. The resulting PCR products were analyzed on a 1% agarose gel and purified by QIAquick gel extraction kit (Qiagen, Germany). A direct sequencing reaction was performed using gene- and strain-specific primers and BigDye terminator cycling at the DNA/Oligonucleotide Synthesis core of Temasek Life Sciences Laboratories, Singapore. Sequences were analyzed using the Lasergene programs (DNAstar, USA).

**Table 2:** Primer pairs used for the amplification of the EV71 P1 region.

Foreward primer	Sequence (5′→3′)	Reverse primer	Sequence (5′→3′)
A 3 fw	GGGCTCCCAGGTCTCCACAC	A 1237 rev	GGTCGGCCCTGAACACTGC
A 1169 fw	AAGCAGGGAAAGGTGAGTTGT	A 3400 rev	GTGGCTAGGTGTCTGTTTAC
B2 1 fw	ATGGGTTCACAAGTGTCTAC	B2 1240 rev	CAGGATCGGCTCTAAACAC
B2 1160 fw	GTTTGATGGAAAGGCTACGA	B2 3420 rev	AATAAACTTGCTAATTTTGTTG
B4 1 fw	ATGGGCTCACAGGTGTCTACTC	B4 1344 rev	GAAAGACCCGGTGAACATAAAAGT
B4 1230 fw	GGCCGACCCTGGAAGAGA	B4 3353 rev	AGATTGCTGGCCGAACTTT
C2 689 utr fw	GACCCTCAACTCAATCAAAC	C2 1511 rev	GCGTGCGCTCTGTAATG
C2 1191 fw	AGCCCAGGCAGGAAAAGGTGAGTT	C2 3369 rev	AACTCACCTTTTCCTGCCTGGGCT
C4 631 utr fw	GCCATCCGGTGTGCAATAGAGCA	C4 1361 rev	ATCTTGCCGGTAGCCATGAAGGAT
C4 1229 fw	GAGCCGATCCTGGGCGGAATG	C4 3361 rev	CGTAAATGGCCCCGGACTGTTGTC

### Construction of mutant viruses by reverse genetics

The genome of B4 wild type virus was first amplified by RT-PCR and subjected to human RNA polymerase I promoter as described in the previous paper [35]. The infectious plasmids containing B4 cDNA (pJET-B4-wt) were sequenced to confirm their authenticity, and transfected into RD cells to generate RG/B4-wt virus. The mutations were introduced into the pJET-B4-wt plasmid by site-directed mutagenesis (Stratagene, USA) using primers (Table 3). For double mutations in pJET-B4-PE59,67LD, pJET-B4-P59L was further mutated by primers B4-E67D-f and B4-E67D-r. The correct mutated plasmids were transfected into RD cells as above to generate the mutants, and designated as RG/B4-P59L, RG/B2-A62D, RG/B2-A62P, RG/B4-E67D, and RG/B4-PE59, 67LD, respectively.

**Table 3:** Primer pairs used for the amplification of the EV71 mutants.

	Foreward primer	Sequence (5′→3′)
pJET-B4-P59L	B4- VP3-P59L-f	GAGGTTAACAATGTACTCACCAATGCCACCAG
pJET-B4-A62D	B4-VP3-A62D-f	CAATGTACCCACCAATGACACCAGTCTGATGGAAAG
pJET-B4-A62P	B4-VP3-A62P-f	CAATGTACCCACCAATCCCACCAGTCTGATGGAAAG
pJET-B4-E67D	B4- VP3-E67D-f	CACCAGTCTGATGGATAGGCTACGATTCCC

	Reverse primer	Sequence (5′→3′)
pJET-B4-P59L	B4- VP3-P59L-r	CTGGTGGCATTGGTGAGTACATTGTTAACCTC
pJET-B4-A62D	B4-VP3-A62D-r	CTTTCCATCAGACTGGTGTCATTGGTGGGTACATTG
pJET-B4-A62P	B4-VP3-A62P-r	CTTTCCATCAGACTGGTGGGATTGGTGGGTACATTG
pJET-B4-E67D	B4- VP3-E67D-r	GGGAATCGTAGCCTATCCATCAGACTGGTG

### Experimental design for passive protection

The animal experiments were conducted with two week old AG129 mice. These mice were obtained from B&K Universal (UK). They were housed and bred under specific pathogen-free conditions in individual ventilated cages. To test the protective efficacy of the antibody, these mice were randomly divided into two groups of 10 mice each. Group 1 mice (prophylactic group) were injected intraperitoneally with the purified mAb 10D3 antibody (0.1 ml in 50% glycerol dissolved in PBS) at a concentration of 10 mg/g of body weight on day one. Group 2 mice (isotype control group) were given an equal amount of purified mouse IgM as an isotype control (eBioscience, USA). These two groups of mice were then subjected to a lethal challenge with 10^7^ plaque forming units (PFU) of EV71 strain HFM 41 (5865/SIN/00009) via the intraperitoneal route (0.4 ml in PBS), 24 h post-injection of the immunoglobulins. Survival rates and clinical scores of the mice were monitored daily till 14 days post-infection. Total limb paralysis was used as criterion for early euthanasia [30], [36].

### Histopathological analysis

Brain samples were collected, fixed in formalin, embedded in paraffin blocks, cut at 5 mm thickness (Leica Microsystems, Germany), and attached to coated glass slides. The slides were stained with hematoxylin and eosin (H&E) and observed under light microscopy.

## Results

### mAb 10D3 is a universal EV71 neutralizing antibody directed against a conformational epitope

To discover novel neutralizing epitopes of EV71, three BALB/c mice were immunized with 100 µL of inactivated EV71-B4 strain virus in a 1∶1 emulsion with adjuvant (Seppic France). The EV71-B4 strain (5865/SIN/000009) was propagated in RD cells, the supernatant containing the virus was inactivated with BEI, and concentrated by ultracentrifugation prior to immunization. Boosters were administered at 14 day intervals and the sera were tested 7 days later for the presence of EV71-specific antibodies by IFA of EV71-infected Vero cells. Once the sera exhibited positive IFA signals, an intraperitoneal booster was administered without adjuvant, and the spleens were harvested three days later. Splenocytes were fused with myeloma cells, the resultant hybridomas were cultured in selective medium, and supernatants were screened by IFA of EV71-B4-infected Vero cells. Positive hybridomas were subcloned by limiting dilution, and their supernatants were analyzed for the presence of neutralizing antibody.

**Figure 1:**
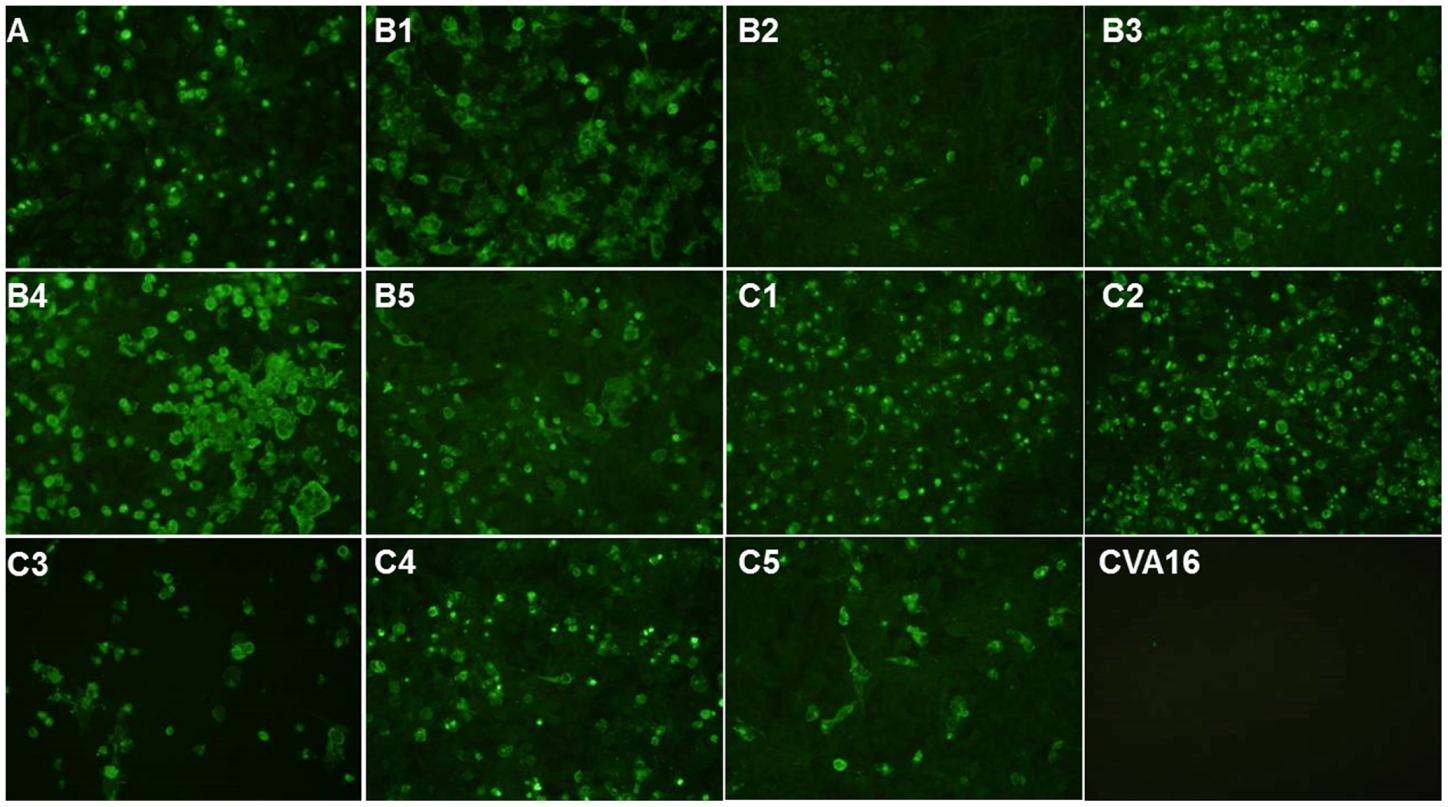
mAb 10D3 recognizes all 11 EV71 subgenogroups and does not cross-react to coxsackievirus A16. IFA of Vero cells infected with heterologous EV71 virus strains and a coxsackievirus A16 (GenBank U05876). Cytopathic effects are visible at 2 days post infection when cells were fixed and labeled with supernatant of mAb 10D3 secreting hybridoma cells. FITC conjugated anti-mouse mAb was used to detect signals. While all 11 EV71 subgenogroups were recognized by mAb 10D3(Parts A, B1, B2, B3, B4, B5, C1, C2, C3, C4, C5), no signal was observed for CVA16(Part CVA16).

**Figure 2:**
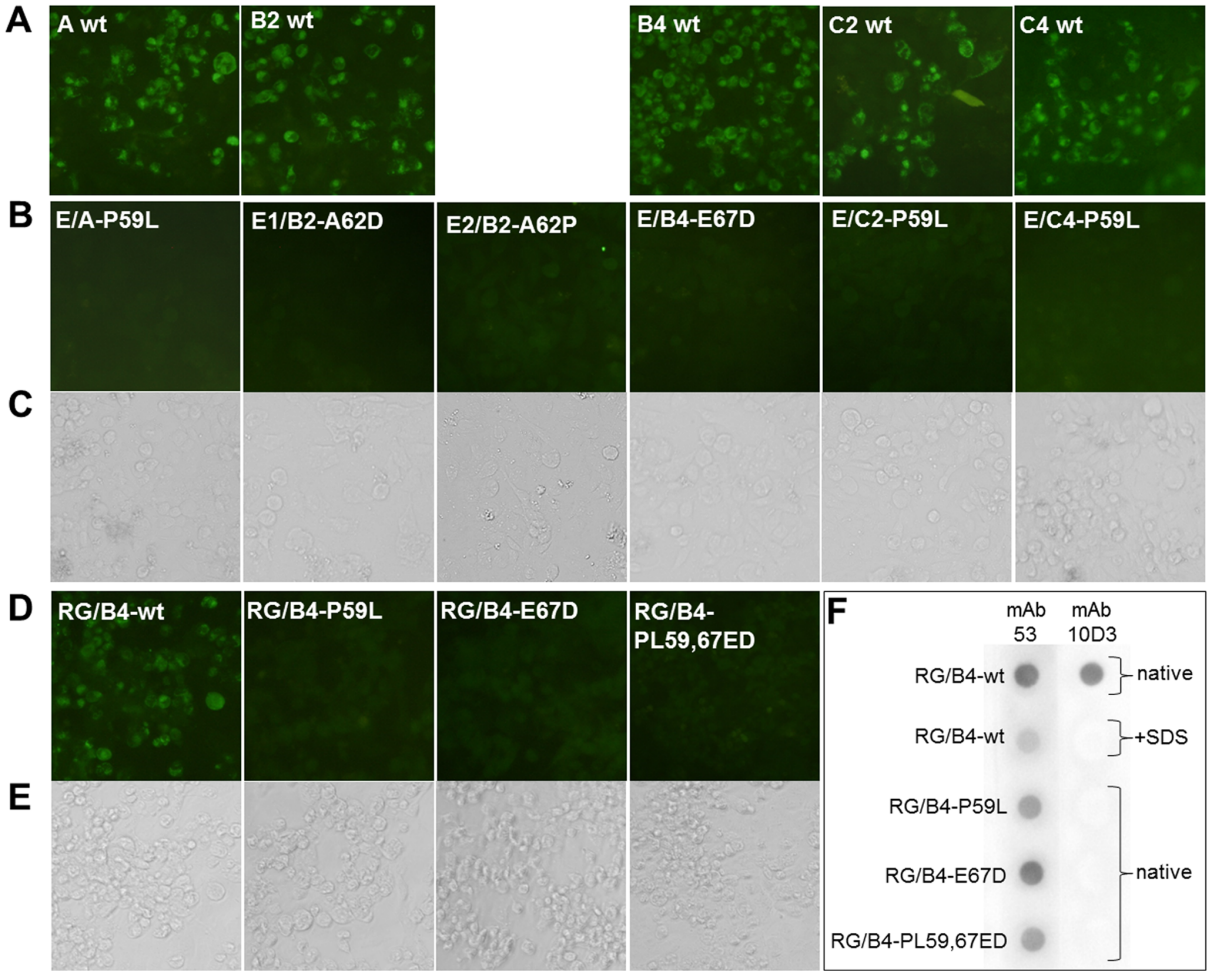
Escape mutations P59L, A62D, A62P or E67D of VP3 protein abolish mAb 10D3 binding. (**A, B, C**) Escape mutants of mAb 10D3 were created by incubating EV71 virus strains A, B2, B4, C2 and C4 with an excess of mAb. Escaped viruses were collected and added to fresh RD cells until CPE was visible (C). Cells were then fixed and labeled with mAb 10D3 followed by FITC coupled secondary antibody. Cells infected with wild-type viruses served as positive controls (A). MAb 10D3 staining was abolished in all escape mutants carrying either the mutation P59L, A62D, A62P or E67D (B). (**D, E**) RG viruses were engineered using the wild-type B4 backbone. The representative escape mutations P59L and E67D were introduced individually (RG/B4-P59L, RG/B4-E67D) or together (RG/B4-PE59,67LD). RD cells were infected with RG/B4-wt and mutated RG viruses for 2 days until CPE was visible (E). Cells were then fixed and labeled with mAb 10D3 followed by FITC coupled secondary antibody. While RG/B4-wt gave a clear IFA signal, the signal was abolished in all three mutated RGVs (D). (**F**) Dot blot of reverse genetically (RG) engineered EV71-B4 viruses. RG/B4-wt viruses were blotted either native or after denaturing with SDS. While mAb 53, which recognizes a linear epitope on VP1, recognized both forms, mAb 10D3 only reacted with the native form. Introduction of the representative escape mutations P59L and E67D into the RG viruses resulted in a loss of recognition by mAb 10D3 when they were blotted in a native form.

In this screen, several mAbs were isolated and further characterized but we focused our attention on mAb 10D3 since this mAb reacted positively with all 11 EV71 subgenogroups by IFA but did not cross-react to CVA16 (Fig. 1). This is a promising feature as the mAb could potentially be applied for differential diagnosis of HFMD to distinguish CVA from EV71 infections. Secondly, the mAb was able to neutralize all EV71 subgenogroups with a neutralization titer of 2^6^ (genogroups A, B) to 2^8^ (genogroup C) against 100 TCID_50_ of wild-type virus(Table 1) by using hybridoma cell supernatant. This universal neutralization ability makes mAb 10D3 an ideal candidate for diagnosis and treatment of EV71 infection. Since Western blot analysis against whole virus and overlapping EV71 P1 polyprotein fragments tagged with GST did not result in any bands with mAb 10D3, we investigated the reactivity of mAb 10D3 with native and denatured viruses in a dot blot assay. Although mAb 10D3 reacted with reverse genetically engineered wild-type B4 virus (RG/B4-wt) blotted in its native form (RG/B4-wt native), it did not recognize virus denatured by boiling with SDS (RG/B4-wt denatured) (Fig. 2F). MAb 53, which recognizes a linear epitope on VP1, was used as a positive control, and could indeed react with both the native and denatured viruses [30]. In conclusion, mAb 10D3 recognizes a conformational epitope. Finally, the mAb 10D3 immunoglobulin isotype was determined as IgM using the mouse monoclonal antibody isotyping kit (Santa Cruz Biotechnology Inc., USA).

### Mutations of amino acids P59L, A62D, A62P or E67D in VP3 capsid protein abolish neutralization of EV71 by mAb 10D3

The epitope of mAb 10D3 was found to be conformational since this mAb did not react with any capsid protein in a Western blot. Hence, the epitope could not be mapped by the conventional fashion of truncated peptides. Therefore epitope mapping of mAb 10D3 was performed by escape mutant selection. Wild-type EV71 viruses from different subgenogroups (A, B2, B4, C2, C4) were incubated with an excess of mAb 10D3 on RD cells. If no CPE was visible after 4 days, supernatants were filtered and added to fresh RD cells. This process was repeated until CPE was evident. 1 to 3 cycles were necessary to isolate escape mutants for all subgenogroups. The escape mutants were designated E/A (EV71-A), E1–2/B2 (two experiments using EV71-B2 virus), E1–3/B4 (three individual experiments using EV71-B4 virus), E/C2 (EV71-C2), and E/C4 (EV71-C4), their TCID_50_ was measured by end-point dilution assay and they were tested for reactivity with mAb 10D3 by IFA. RD cells were infected with an equal amount of either wild-type virus as positive controls or escaped viruses and observed for 2 days until CPE was visible (Fig. 2C). After incubation with mAb 10D3, there was a clear fluorescent signal for the wild-type viruses (Fig. 2A), but no signal was detected for any of the corresponding escape mutants (Fig. 2B). To further confirm that the escape mutants have evaded mAb 10D3 binding, a microneutralization assay against 100 TCID_50_ of escaped viruses was conducted. There was no more virus neutralization by mAb 10D3 of any of the identified escape mutants. However, neutralizing mAb 51, which recognizes an unaltered epitope on VP1, was able to neutralize all escape mutants (Table 4).

**Table 4:** Escape mutations and neutralization titers against homologous and heterologous Mabs.

Subgeno-group	Name	Description	VP3 Mutation	Neutralization Titer Mab 10D3	Neutralization Titer Mab 51
A	A-wt	Wild-type virus		2∧6	2∧10
	E/A	Escape mutant	P59L	<2∧1	2∧10
B2	B2-wt	Wild-type virus		2∧6	2∧10
	E1/B2	Escape mutant	A62D	<2∧1	2∧10
	E2/B2	Escape mutant	A62P	<2∧1	2∧10
	RG4/B4-A62D	Mutated RG virus	A62D	<2∧1	2∧10
	RG5/B4-A62P	Mutated RG virus	A62P	<2∧1	2∧10
B4	B4-wt	Wild-type virus		2∧6	2∧10
	E1/B4	Escape mutant	E67D	<2∧1	2∧10
	E2/B4	Escape mutant	E67D	<2∧1	2∧10
	E3/B4	Escape mutant	E67D	<2∧1	2∧10
	RG/B4-wt	RG virus		2∧6	2∧10
	RG1/B4-P59L	Mutated RG virus	P59L	<2∧1	2∧10
	RG2/B4-P59L	Mutated RG virus	E67D	<2∧1	2∧10
	RG3/B4-PE59,67LD	Double mutant	P59L+E67D	<2∧1	2∧10
C2	C2-wt	Wild-type virus		2∧8	2∧9
	E/C2	Escape mutant	P59L	<2∧1	2∧9
C4	C4-wt	Wild-type virus		2∧8	2∧9
	E/C4	Escape mutant	P59L	<2∧1	2∧9

Microneutralization assays were conducted with 100 TCID50 of wild-type viruses, escape mutants and mutated RG viruses against the Mab 10D3 and VP1 linear neutralizing Mab 51 (concentrated, 1 mg/ml) as a positive control.

To delineate the amino acid mutations associated with neutralization escape of the different subgenogroups, the P1 structural gene region of each escape mutant was sequenced and compared to its parental strain. In the eight escape mutants, four mutations were identified in the structural gene VP3. The mutants E1–3/B4 derived from the parental strain B4 harbored a glutamate to aspartate substitution at amino acid position 67 of VP3, while the other three mutants E/A, E/C2, E/C4 derived from A, C2, and C4 subgenogroups carried a proline to leucine substitution at amino acid position 59 of VP3. Two separate mutations were discovered at amino acid 62 of the escape mutants from the B2 strain: an alanine to aspartic acid or proline (Table 5).

**Table 5:** Alignment of EV71 VP3 protein from subgenogroups and escape mutants.

R	N	L	L	E	L	C	Q	V	E	T	I	L	E	V	N	N	V	P	T	N	A	T	S	L	M	E	R	L	R	F	P	V	S	A	Q	A	G	K	G	Majority
								50										60										70										80		
R	N	L	L	E	L	C	Q	V	E	T	I	L	E	V	N	N	V	P	T	N	A	T	S	L	M	E	R	L	R	F	P	V	S	A	Q	A	G	K	G	A BrCr VP3.pro
R	N	L	L	E	L	C	Q	V	E	T	I	L	E	V	N	N	V	P	T	N	A	T	S	L	M	E	R	L	R	F	P	V	S	A	Q	A	G	K	G	B2 7423-MS-87 VP3.pro
R	N	L	L	E	L	C	Q	V	E	T	I	L	E	V	N	N	V	P	T	N	A	T	S	L	M	E	R	L	R	F	P	V	S	A	Q	A	G	K	G	B4 5865-SIN-000009 VP3.pro
R	N	L	L	E	L	C	Q	V	E	T	I	L	E	V	N	N	V	P	T	N	A	T	S	L	M	E	R	L	R	F	P	V	S	A	Q	A	G	K	G	B5_NUH0083_VP3.pro
R	N	L	L	E	L	C	Q	V	E	T	I	L	E	V	N	N	V	P	T	N	A	T	S	L	M	E	R	L	R	F	P	V	S	A	Q	A	G	K	G	C2 NUH007-SIN-08-VP3.pro
R	N	L	L	E	L	C	Q	V	E	T	I	L	E	V	N	N	V	P	T	N	A	T	S	L	M	E	R	L	R	F	P	V	S	A	Q	A	G	K	G	C4yama lab strain VP3.pro
R	N	L	L	E	L	C	Q	V	E	T	I	L	E	V	N	N	V	P	T	N	A	T	S	L	M	***D***	R	L	R	F	P	V	S	A	Q	A	G	K	G	E1 10D3+B4 VP3.pro
R	N	L	L	E	L	C	Q	V	E	T	I	L	E	V	N	N	V	P	T	N	A	T	S	L	M	***D***	R	L	R	F	P	V	S	A	Q	A	G	K	G	E2 10D3+B4 VP3.pro
R	N	L	L	E	L	C	Q	V	E	T	I	L	E	V	N	N	V	P	T	N	A	T	S	L	M	***D***	R	L	R	F	P	V	S	A	Q	A	G	K	G	E3 10D3+B4 VP3.pro
R	N	L	L	E	L	C	Q	V	E	T	I	L	E	V	N	N	V	***L***	T	N	A	T	S	L	M	E	R	L	R	F	P	V	S	A	Q	A	G	K	G	E4 10D3+A VP3.pro
R	N	L	L	E	L	C	Q	V	E	T	I	L	E	V	N	N	V	***L***	T	N	A	T	S	L	M	E	R	L	R	F	P	V	S	A	Q	A	G	K	G	E5 10D3+C2 VP3.pro
R	N	L	L	E	L	C	Q	V	E	T	I	L	E	V	N	N	V	***L***	T	N	A	T	S	L	M	E	R	L	R	F	P	V	S	A	Q	A	G	K	G	E6 10D3+C4 VP3.pro
R	N	L	L	E	L	C	Q	V	E	T	I	L	E	V	N	N	V	P	T	N	***D***	T	S	L	M	E	R	L	R	F	P	V	S	A	Q	A	G	K	G	E7 10D3+B2 VP3.pro
R	N	L	L	E	L	C	Q	V	E	T	I	L	E	V	N	N	V	P	T	N	***P***	T	S	L	M	E	R	L	R	F	P	V	S	A	Q	A	G	K	G	E8 10D3+B2 VP3.pro
R	N	L	L	E	***I***	C	***R***	V	E	T	I	L	E	V	N	N	***L***	***Q***	***S***	N	***E***	T	***T***	P	M	***Q***	R	L	***C***	F	P	V	S	***V***	Q	***S***	***K***	***T***	G	CA16 U05876 VP3.pro

Partial alignment of the VP3 protein sequences of EV71 strains from representative subgenogroups, escape mutants, and coxsackievirus A16. The four escape mutations are highlighted in the boxes. While the EV71 strains showed 100% homology in the shown VP3 region, the amino acid sequence of CVA16 was distinctly different. The differences were shown by italic and bold.

### Introduction of VP3 mutations into reverse genetically engineered EV71-B4 virus mimics the escape mutant phenotype

**Figure 3:**
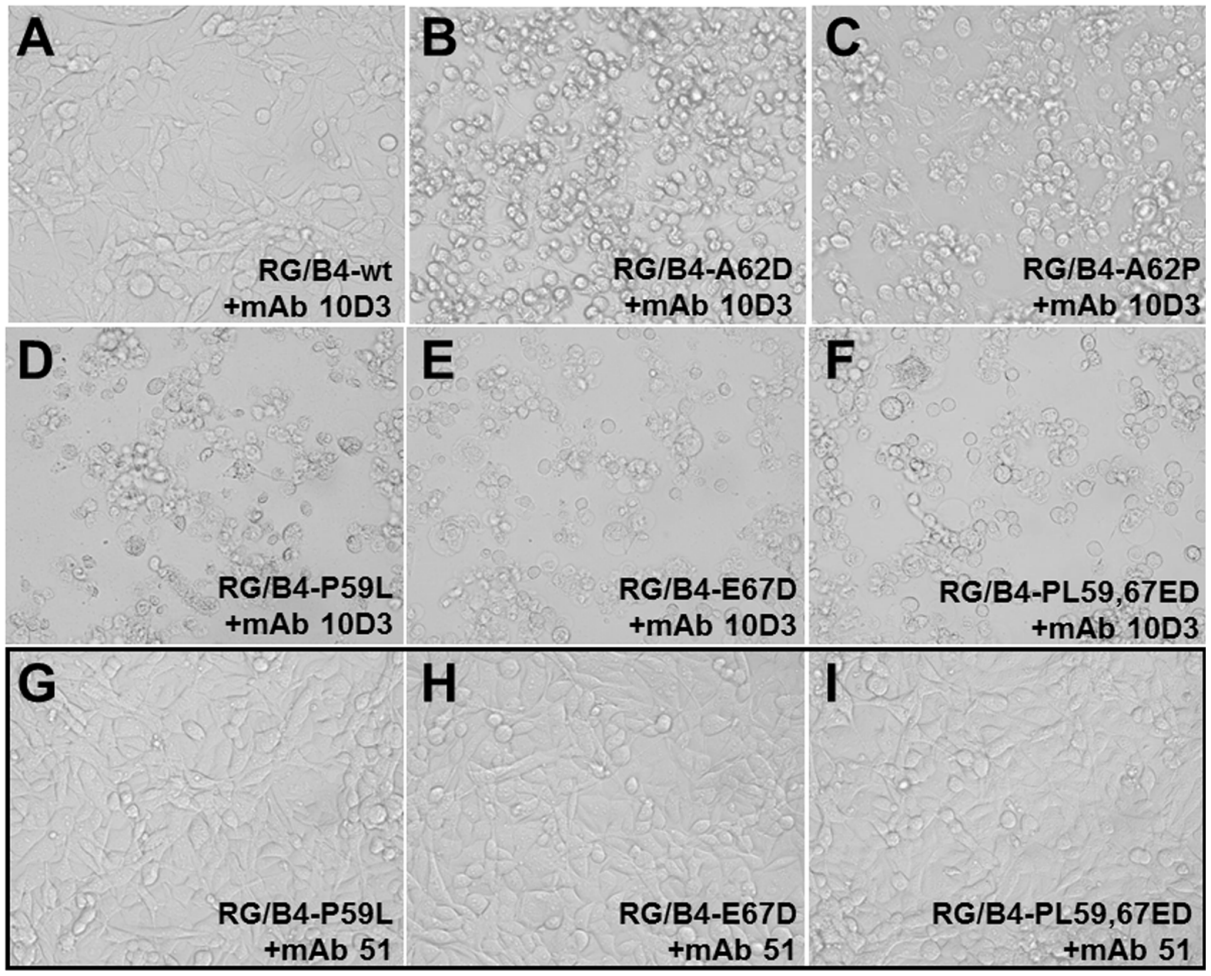
P59L, A62D, A62P or E67D mutation of VP3 protein impedes mAb 10D3 mediated virus neutralization. The loss of neutralization of mutated RG viruses was established in a microneutralization assay. 100 TCID50 of B4 RGV and mutated RGVs were mixed with serial dilutions of mAb 10D3 or positive control mAb 51. While 10D3 neutralized the B4 RGV efficiently (A), no neutralization was observed for the mutated RGVs (B–F), indicating that the P59, A62D, A62P and E67 of VP3 are sufficient for the abolishment of virus neutralization by mAb 10D3. The positive control mAb 51, which recognizes a conserved linear epitope on VP1, efficiently neutralized all RGVs (representative RGVs are shown in G–I).

Since we have discovered only a single amino acid mutation in the capsid proteins of each escape mutant, it can be inferred that these residues are essential for both mAb 10D3 binding and virus neutralization. To test this hypothesis, we engineered an EV71-B4 virus consisting of the EV71-B4 (5865/SIN/000009) sequence by utilizing a human RNA polymerase I driven reverse genetics system [35]. The four VP3 mutations P59L, A62D, A62P and E67D were then introduced alone (RG/B4-P59L, RG/B4-A62D, RG/B4-A62P, RG/B4-E67D) or in tandem (RG/B4-PE59, 67LD) into the wild-type RG virus (B4 RGV) by site-directed mutagenesis. The RG viruses were then rescued in RD cells and passage 2 viruses were used in subsequent experiments. The binding ability of mAb 10D3 to the representative mutated RG viruses RG/B4-P59L, RG/B4-E67D and RG/B4-PE59,67LD was first tested by IFA (Fig. 2DE), and dot blot (Fig. 2F). RD cells were infected with RG/B4-wt virus as positive control or the mutated RG viruses. The cells were fixed 2 dpi when CPE was clearly observed (Fig. 2E). While the original virus (RG/B4-wt) was clearly detected by mAb 10D3, no fluorescence was visible for the mutated RGVs carrying either a single or double mutations. Additionally, mAb 10D3 was unable to neutralize the mutated RG viruses (RG/B4-P59L, RG/B4-A62D, RG/B4-A62P, RG/B4-E67D, RG/B4-PE59,67LD) by an *in vitro* microneutralization assay, while the neutralization titer of mAb 10D3 against RG/B4-wt reached 2^6^ which was the same as for B4-wild-type (Table 4). As a positive control, mAb 51 against the linear neutralizing epitope KQEKD on VP1 was incubated with the mutated RG viruses. Since the VP1 epitope was unaffected by our mutagenesis, mAb 51 was still able to efficiently neutralize all RG viruses (Fig. 3). Hence we have demonstrated that the four escape mutations (P59L, A62D, A62P and E67D) are sufficient for the abolishment of mAb 10D3 binding to the VP3 protein and neutralization of EV71 virus.

### The “knob” of VP3 forms a conserved conformational epitope of EV71

Having identified 3 amino acids of VP3 that are essential for mAb 10D3 binding and neutralization, we next investigated whether these residues are conserved in all of the fully sequenced EV71 strains available on GenBank. BLAST analysis of amino acids 59–67 of VP3 revealed a total of 388 EV71 hits which were all 100% identical in the region analyzed, while the amino acid identity was 97% for the full VP3 protein. VP3 is thus more highly conserved between subgenogroups than VP1 (93% identity), making it an ideal target for a diagnostic or therapeutic mAb. The same region was also compared to CVA16 strains, which exhibited no sequence homology to EV71 (Table 5).

**Figure 4:**
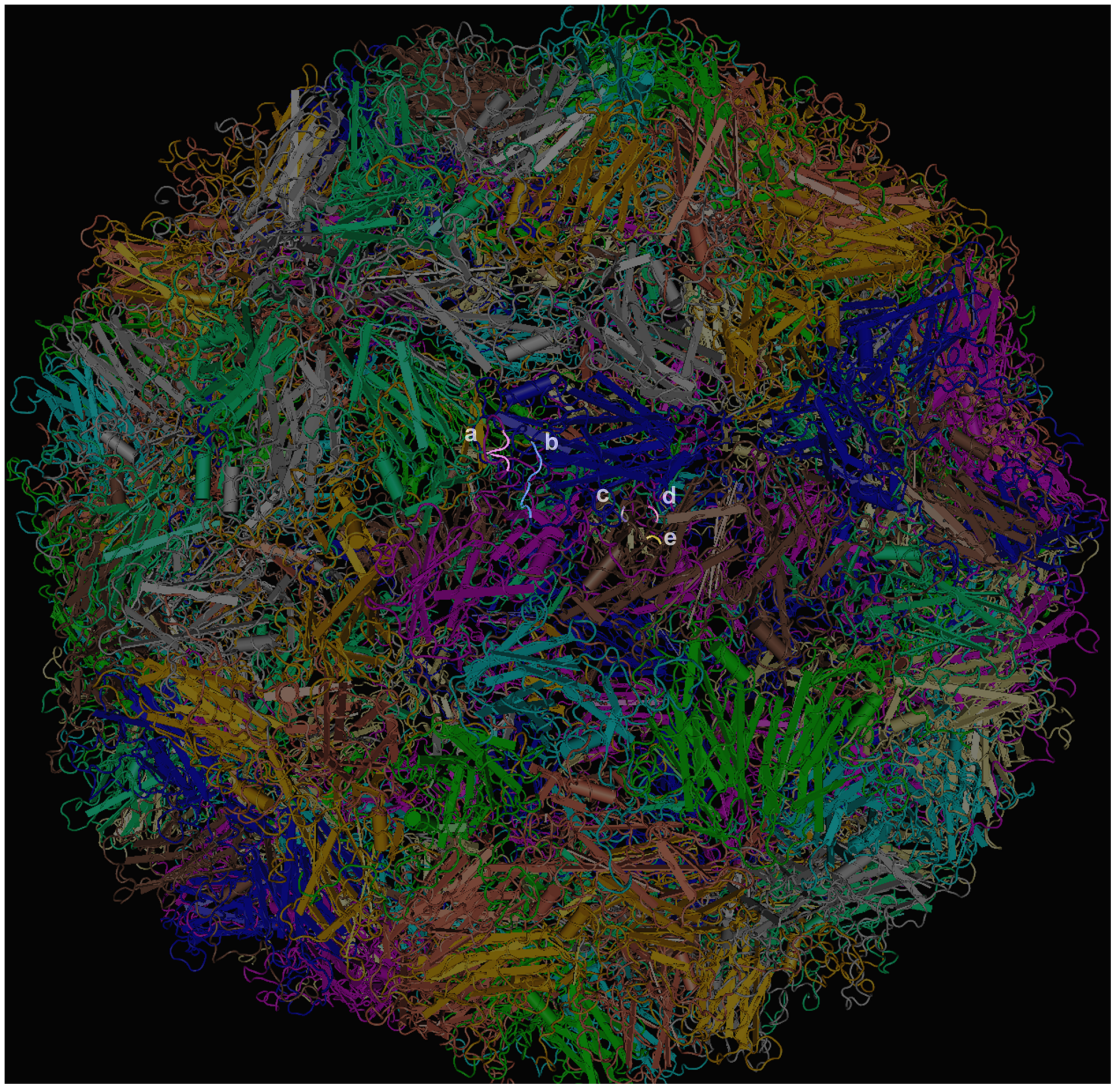
Location of the epitopes of mAb 10D3, 7C7 and 51 on the surface of EV71 whole virus particle. The 3D crystal structure of EV71 C4 virus (MMDB ID: 97658) was downloaded from NCBI and viewed in the Cn3D program. The mapped epitopes of EV71 specific mAbs 51 (VP1 215-KQEKD-219) and 7C7 (VP2 142-EDSHP-146) as well as the three escape mutation sites of mAb 10D3 at amino acid positions 59, 62 and 67 on VP3 are indicated. a: 51 (VP1 215-KQEKD-219) (grey), b: 7C7 (VP2 142-EDSHP-146) (blue), c: 10D3 62A (purple), d: 10D3 67E(pink), e: 10D3 59P(yellow).

**Figure 5:**
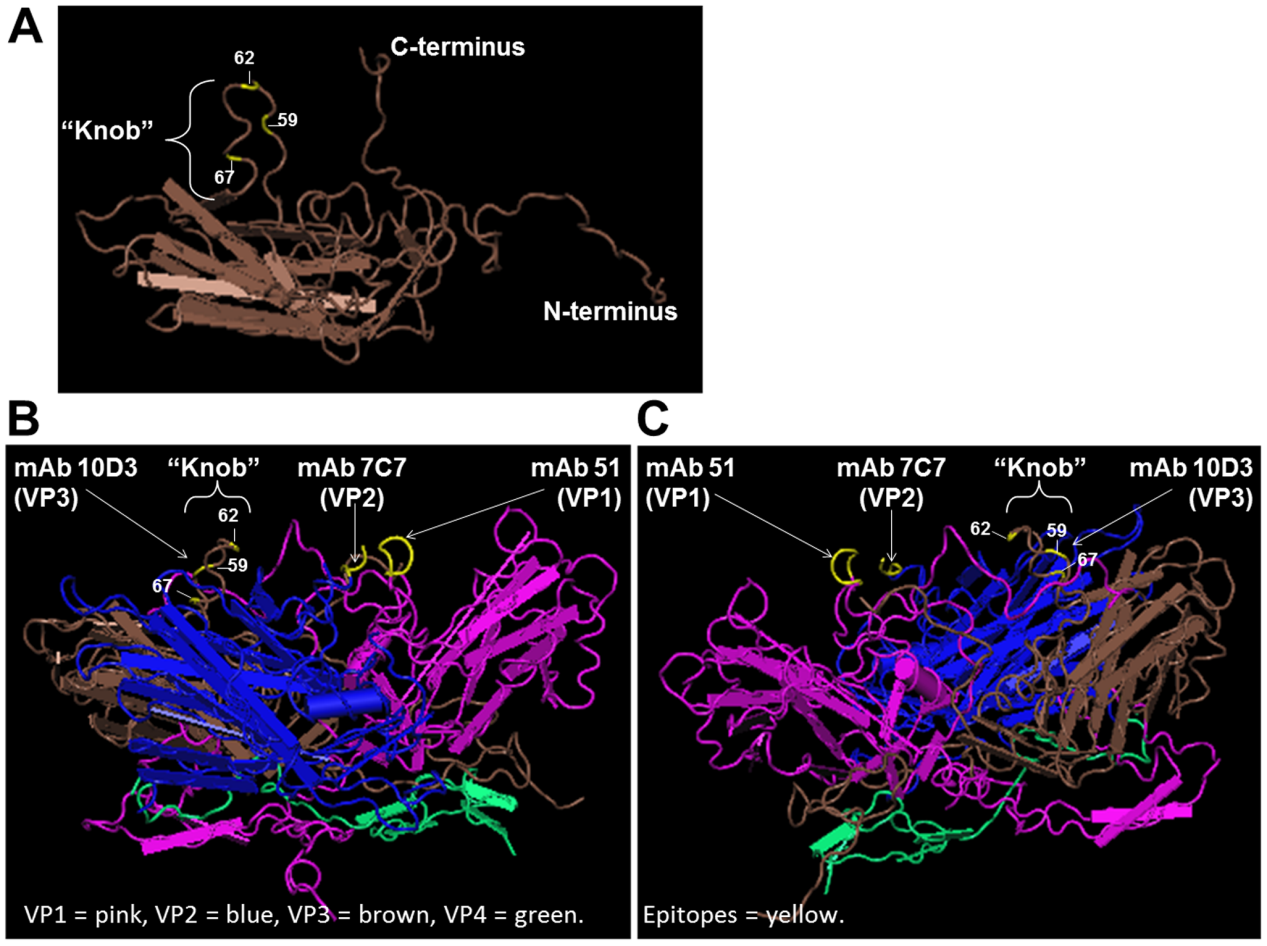
Stereographic images of 10D3 escape mutations. The 3D crystal structure of EV71 C4 virus (MMDB ID: 97658) was downloaded from NCBI and viewed in the Cn3D program. (**A**) VP3 protein. The EV71 VP3 protein is shown in a side on view with the outside of the virion located at the top of the image and the inside on the bottom. The “knob” structure is indicated with the bracket. It consists of a helical protrusion on the capsid surface encompassing amino acids 55 to 69 of VP3. The three escape mutation sites of residues 59, 62 and 67 are indicated in yellow. (**B, C**) EV71 protomer with mAb epitopes. A protomer of the virus capsid is shown, containing one of each of the viral capsid proteins VP1 (pink), VP2 (blue), VP3 (brown), and VP4 (green). The top of the image corresponds to the surface of the virion and the bottom to the inside. The mapped epitopes of EV71 specific mAbs 7C7 (VP2 142-EDSHP-146) and 51 (VP1 215-KQEKD-219) as well as the three escape mutation sites of mAb 10D3 at amino acid positions 59, 62 and 67 on VP3 are indicated in yellow (arrows). Two orientations are shown: (**B**) The major groove, formed by VP1, is visible to the right while VP2 is in the foreground and VP3 in the back. (**C**) The image has been rotated to show the positions of the escape mutations on VP3 more clearly. VP1 and VP3 are now in the foreground and VP2 in the back.

**Figure 6:**
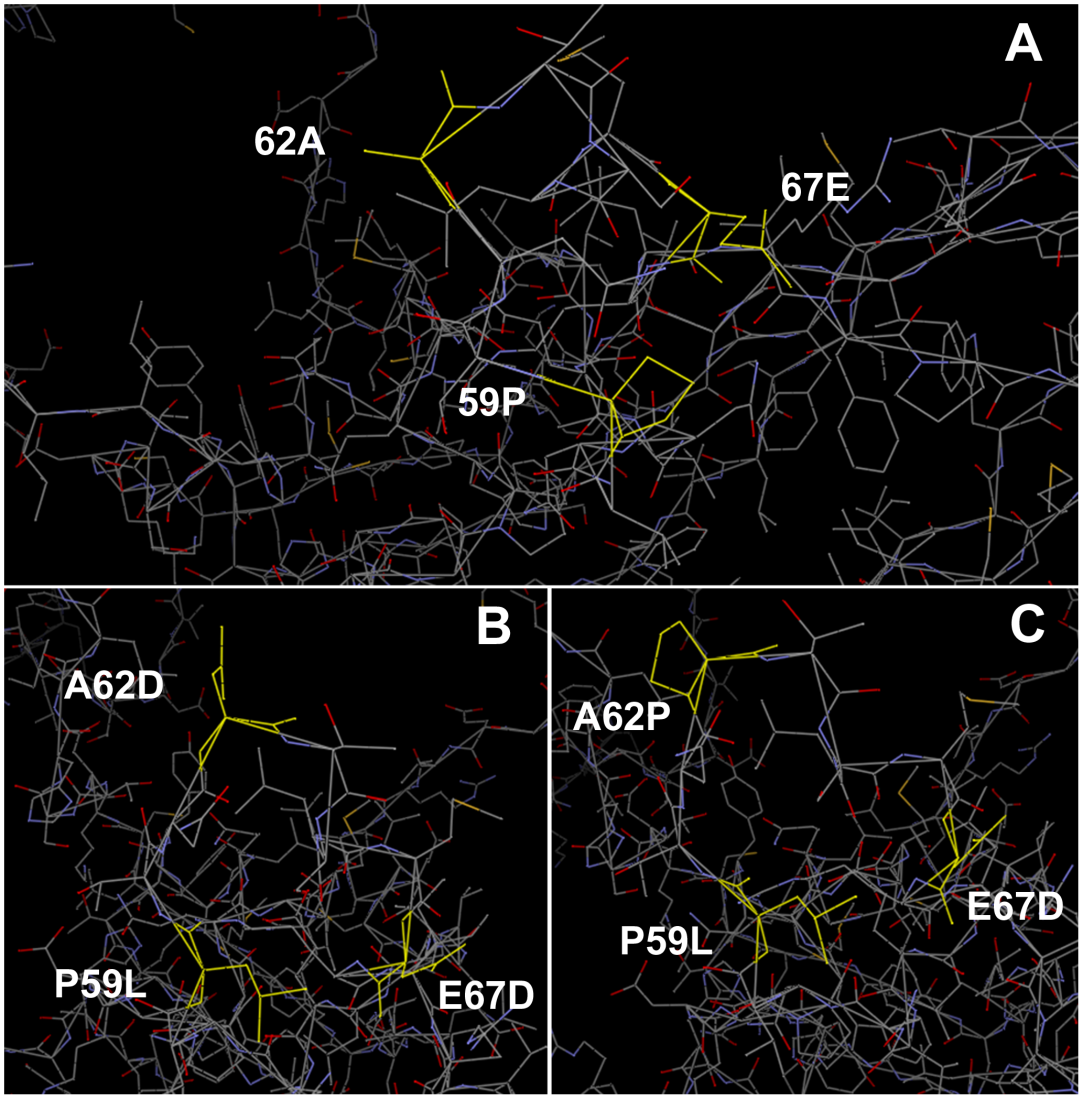
Stereographic images of 10D3 escape mutations. (**A**) The 3D structure of the VP3 protein of the EV71 B4 virus is depicted. The amino acids 59P, 62A, and 67E are shown in yellow. (**B, C**) The escape mutations P59L, A62D, A62P, and E67D are illustrated in yellow. The images have been generated by Swiss Model (http://beta.swissmodel.expasy.org [51]–[53]) and were analyzed by Vector NTI v11.5.1 (Life Technologies).

In the view of the recently available 3D crystal structure of EV71-C4, the epitope of mAb 10D3 could be located by stereographic imaging [17], [18]. To analyze the location of the VP3 epitope in relation to previously identified EV71 epitopes (the linear neutralizing epitope KQEKD of mAb 51 on VP1, and the linear epitope EDSHP of mAb 7C7 on VP2) [30], [37], we studied stereographic images of EV71 protomers. In Fig. 4 the epitopes of mAb 51, 7C7 and 10D3 are shown on the virus surface. In Fig. 5 and 6 the EV71 VP3 protein is shown and the escape mutations P59L, A62D, A62P and E67D are indicated. Both sites lie on the major protrusion of VP3 on the capsid surface termed “knob” [18]. In Fig. 5B and 5C, a protomer consisting of one copy each of the viral capsid proteins VP1 (pink), VP2 (blue), VP3 (brown) and VP4 (green) is shown. The top of the image corresponds to the surface of the virion and the bottom (where VP4 is located) to the inside. Two orientations are shown: (B) The major groove, formed by VP1, is visible to the right, while VP2 is in the foreground and VP3 in the back. (C) The image was rotated to display the positions of the escape mutations on VP3 more clearly. VP1, VP3 are now in the foreground and VP2 in the back. Indicated in yellow are the epitopes of some previously identified mAbs of EV71 as well as the conformational neutralizing epitope of mAb 10D3 in the knob region of VP3 (arrows). The three escape mutation sites at amino acid positions 59, 62 and 67 on VP3 are indicated. As shown in Fig. 6, the conformational changes generated by the escape mutants were as follows: A loss of the cyclic structure of proline's side chain in the P59L mutant, the addition of a cyclic side chain in the A62P mutant, or of a carboxyl group in the A62D mutant, and the loss of a methylene bridge in the E67D mutant.

### Passive protection of AG 129 mice against a lethal EV71 strain

**Figure 7:**
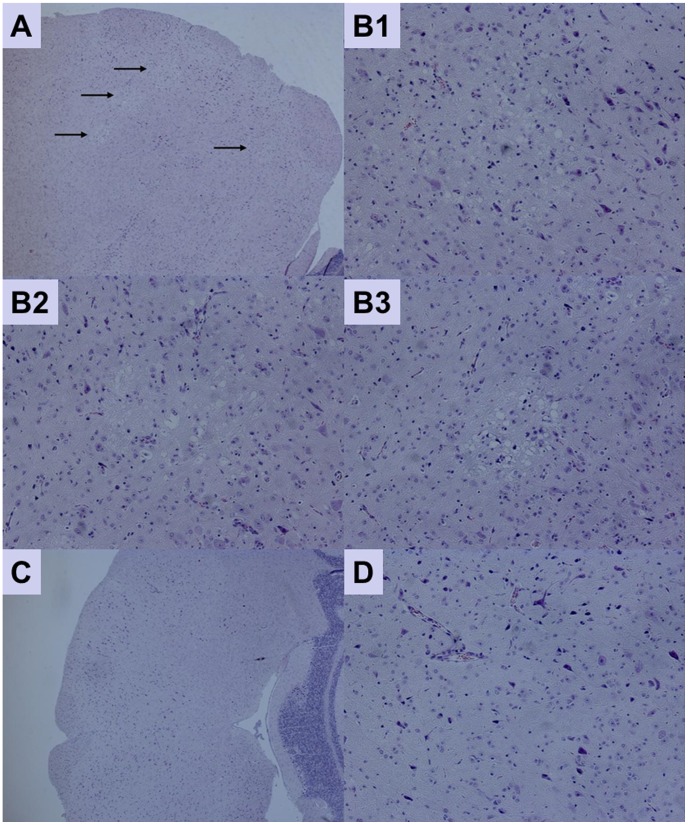
Brain histopathology of EV71 infected AG129 mice and of mice prophylactically protected with mAb 10D3. (**A**) Cross-section of brain stem from isotype control mice with arrows pointing to regions with neuropil vacuolation and neuronal loss without inflammation in the brain stem (at original magnification of 40×). (**B1–B3**) Higher power view of Fig. 7A at original magnification of 200×. (**C**) Cross-section of brain stem from mice prophylactically protected with mAb 10D3 showing no significant pathology (at original magnification of 40×). (**D**) Higher power view of Fig. 7C at original magnification of 200×.

To test the protective efficacy of mAb 10D3, two week old AG129 mice were injected intraperitoneally with purified mAb 10D3 or isotype control mAb. One day later, they were challenged with a lethal dose of the virulent EV71 strain HFM41, and clinical scores as well as survival rates of the mice were monitored daily. In the control animals, which received an isotype antibody, 80% developed severe limb paralysis as early as day 6 post-infection. In contrast, the mice pre-treated with mAb 10D3 did not display any of the disease manifestations, and remained healthy throughout the experiment. Our result thus suggested that the anti-EV71 antibody mAb 10D3 (administered at a dose of 10 mg/g of body weight) was able to achieve 100% protection against the lethal EV71 challenge. To confirm the protective efficacy of mAb 10D3, a histopathologic examination of the mouse brains was conducted. Mice from the isotype control group exhibited neuronal vacuolation and neuronal loss without inflammation in the brain stem (Figs. 7A, 7B1, 7B2 and 7B3). In contrast, we did not observe such pathologic changes in mice from the prophylactic group treated with 10D3. The intact brain morphology (Figs. 7C and 7D) suggested that mAb 10D3 was capable of conferring *in vivo* passive protection against EV71 infection.

## Discussion

Despite the evidence that enteroviruses have major conformational neutralization sites on all capsid proteins, to date the only mapped universal neutralization epitope of EV71 is a linear epitope spanning amino acids 215–219 of VP1 [30], [38], whereas the only known conformational epitope is strain-specific and includes amino acid 145 of VP1 [39]. In order to find an optimal mAb for future use as an immunologic therapeutic, either alone or in conjunction with mAb 51, a larger pool of universally neutralizing mAb candidates would be desired. We therefore undertook the task of isolating such mAbs derived from mice immunized with EV71-B4 (5865/SIN/000009). This strain was selected for its virulence, as it consistently attained a higher TCID_50_ in cell culture and was able to cause disease in older mice pups than any other strain we tested (unpublished observations). This strain was isolated from a fatal case of HFMD with encephalitis during the Singapore outbreak of EV71 in 2000 [40]. We found that mAb 10D3 that efficiently neutralized all EV71 subgenogroups without cross-reaction to CVA16, making it a highly specific antibody with a potential application in differential diagnosis of HFMD. Since the epitope of 10D3 was found to be conformational, it was crucial to generate escape mutants in order to map the exact epitope. Mutations of amino acids proline to leucine at position 59, alanine to aspartic acid or proline at position 62, or glutamate to aspartate at position 67 of VP3 resulted in escape from mAb binding and neutralization. These residues are integral to the “knob” structure of EV71 VP3 protein that protrudes out of the capsid surface, and is completely conserved among all EV71 sequences deposited in GenBank. Hence, we have discovered an additional conformational epitope of EV71, and provided the first evidence of another capsid protein involved in EV71 virus neutralization besides VP1.

The knob of EV71 VP3 encompasses residues 55 to 69 of VP3 - making it longer than the knob described for coxsackievirus B3 (CVB3) - and contains a major neutralization site for other picornaviruses such as CVB3, poliovirus 1, human rhinovirus 14 and hepatitis A virus [41]–[44]. In hepatitis A virus the immunodominant epitope involves residue 70 of VP3 which is close to the mutations identified in our screen [44]. The situation is more complex for poliovirus: while N-AgI is the major neutralizing epitope for the PV-3 serotype (Sabin), N-AgII and N-AgIII are immunodominant for PV-1 (Mahoney) [45]. However, neutralizing IgA mAbs, derived from both PV-1 and PV-3 immunized mice, were all predominantly directed against the N-AgIII epitope [46], [47] which is formed by amino acids 58–59 of VP3 and 286–290 of VP1 [31]. Mapped onto the EV71 virus structure, these residues are in close proximity of the VP3 knob and pass right in front of (Fig. 5B pink tube at the upper right of the protomer structure). It remains to be seen if some 10D3 escape mutants might also have alterations in the C-terminus of VP1 in addition to the VP3 knob, as these residues may contribute to epitope formation.

Escape mutants of another enterovirus, coxsackievirus B3, harbor mutations in both the knob of VP3 and the puff of VP2. The VP3 mutation was mapped to residue 60, while the VP2 mutation was located on residue 158. Stereographic imaging revealed that the two mutations lie in close proximity to one another, forming a conformational epitope [41]. By analogy to CVB3, we more closely analyze the other identified epitopes of EV71. Since our previously identified, non-neutralizing, linear mAb 7C7 against EV71 VP2 has a linear epitope quite close to CVB3 VP2 158, i.e. residues 142–146 [37], we investigated whether these amino acids might be involved in forming a conformational epitope with VP3 in EV71 as well. As can be readily deduced from the stereographic images, the VP2 puff of EV71 resides much further away from the VP3 knob, and neither residues 142–146 nor 158 are in close proximity to our identified escape mutations. Instead, the VP2 epitope 142-EDSHP-146 is adjacent to the neutralizing VP1 epitope 215-KQEKD-219 indicating that these two linear epitopes may interact in EV71 which could explain the low (<1∶14) neutralizing activity observed for the commercially available mAb979 (Merck Millipore, Germany) which recognizes a peptide of VP2 spanning residues 136–150 which encompasses the 7C7 epitope [48].

Neutralizing monoclonal antibodies are specific antiviral agents that can be used for the passive immunization of patients with acute viral infections. They offer a selective advantage over pooled human sera that are more commonly used in IVIG treatment by reducing the risk of transmitting pathogens, and by alleviating batch-to-batch variability, availability of donors, and the presence of non-neutralizing antibodies. Several factors have to be considered when using mAbs instead of polyclonal serum for IVIG, including (a) the antigenic variability of circulating strains, i.e. the mAb must cross-neutralize all existing subtypes to be useful; (b) The risk of escape mutations, e.g. mutants may emerge under selective pressure such as the presence of a neutralizing antibody. To circumvent this risk, a cocktail of two antiviral mAbs against non-overlapping epitopes can be administered, where escape mutation from a single mAb does not interfere with the neutralizing capability of the second mAb. A combination of synergistic mAbs may also reduce the required dosages [49], [50]. In conclusion, the protective efficacy of mAb 10D3 was evaluated and verified by an animal challenge experiment using a lethal dose of EV71. All mice prophylactically treated with mAb 10D3 survived the lethal challenge without showing any disease symptoms. Hence, mAb 10D3 holds promise for being further developed as a prophylactic agent against EV71-associated HFMD.
